# Targetoid Hemosiderotic Hemangioma With Spontaneous Remission and Recurrence in a Male Patient: A Case Report

**DOI:** 10.7759/cureus.67339

**Published:** 2024-08-20

**Authors:** Mridul Bhardwaj, Revat J Meshram, Atharv Sardesai, Dev B Goel

**Affiliations:** 1 Dermatology, Jawaharlal Nehru Medical College, Datta Meghe Institute of Higher Education and Research, Wardha, IND; 2 Paediatrics, Jawaharlal Nehru Medical College, Datta Meghe Institute of Higher Education and Research, Wardha, IND

**Keywords:** hemangioma, ecchymotic ring, papule, recurrent, hemosiderotic

## Abstract

Targetoid hemosiderotic hemangioma is a rare, characteristic, benign lymphovascular lesion that usually affects the trunk and lower limbs. It has a diverse clinical appearance. Most affected people are in their 20s. A violaceous solitary papule encircled by a pale, narrow region and an ecchymotic ring peripherally, mimicking a target, is the most prevalent clinical characteristic. In many cases, the reported dermoscopic signs and patterns of hemosiderotic hemangioma have been found to be adequate for establishing a clinical diagnosis. The following is a case of a 23-year-old male presenting with blue-black vascular lesions on the lateral aspect of the right deltoid with a red ecchymotic ring. The 2×1 cm-sized lesion has a history of spontaneous remission and reappearance. Based on dermoscopic findings, a diagnosis of targetoid hemosiderotic hemangioma was made.

## Introduction

All vascular tumors with hobnail-like endothelial cells on histology, whether benign or malignant, are collectively referred to as hobnail hemangiomas. This group includes retiform hemangioendothelioma, polymorphic hemangioendothelioma, progressive lymphangioma, skin or soft tissue angiosarcoma, Dabska tumor (malignant endovascular papillary angioendothelioma), and unusual vascular lesions following radiotherapy. Targetoid hemosiderotic hemangioma (THH) was initially identified by Aronberg and Santa Cruz in the 1980s as a hobnail hemangioma. Its histology and morphology differ according to the evolutionary stage [1]. Usually, it is seen in the limbs or trunk. It can manifest at any age between 5 and 67 years old, with younger age groups experiencing it more frequently. There is no preference for either gender [2]. Trauma to an underlying hemangioma and hormonal factors, as demonstrated by alterations in the lesions in women during menstruation, account for the pathophysiology of THH [3]. Hobnail hemangioma is another name for THH, a benign vascular tumor with a targetoid form. The clinical lesion known as THH appears as a tiny violaceous papule encircled by a peripheral ecchymotic ring and a pale, thin region. Mostly, such lesions present with a diameter not exceeding more than 1 cm [4]. It is possible that the patient's presentation will not always be as distinctive as in this particular instance. Lesions may appear as a papule or angiomatous macule without a halo. This variety has been explained by several theories, including distinct stages in the lesion's genesis and devolution, hormonal effects, or trauma to an already-existing angioma. Spontaneous devolution is shown by pale regions within the purpuric ring [5].

## Case presentation

**Figure 1:**
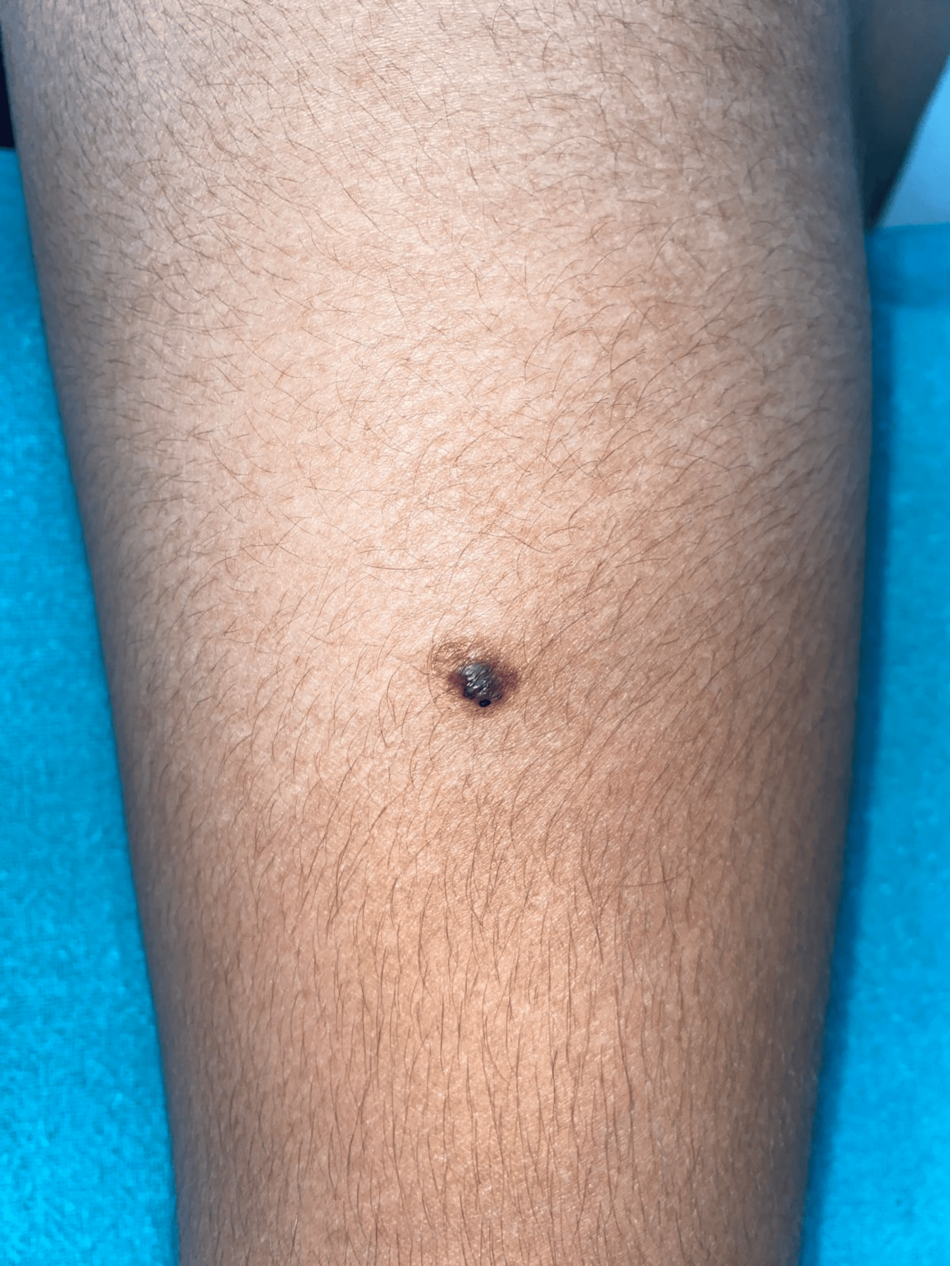
A blue-black lesion surrounded by a red ecchymotic ring on the right arm

A 23-year-old male from Uttar Pradesh presented to the outpatient department with an asymptomatic vascular lesion on the deltoid region of his right arm for the past eight years. The lesion was blue-black, surrounded by a red ecchymotic ring. The lesion measured 2x1 cm and presented with a history of spontaneous resolution along with reappearances after two to three months of resolution (Figure 1).

**Figure 2:**
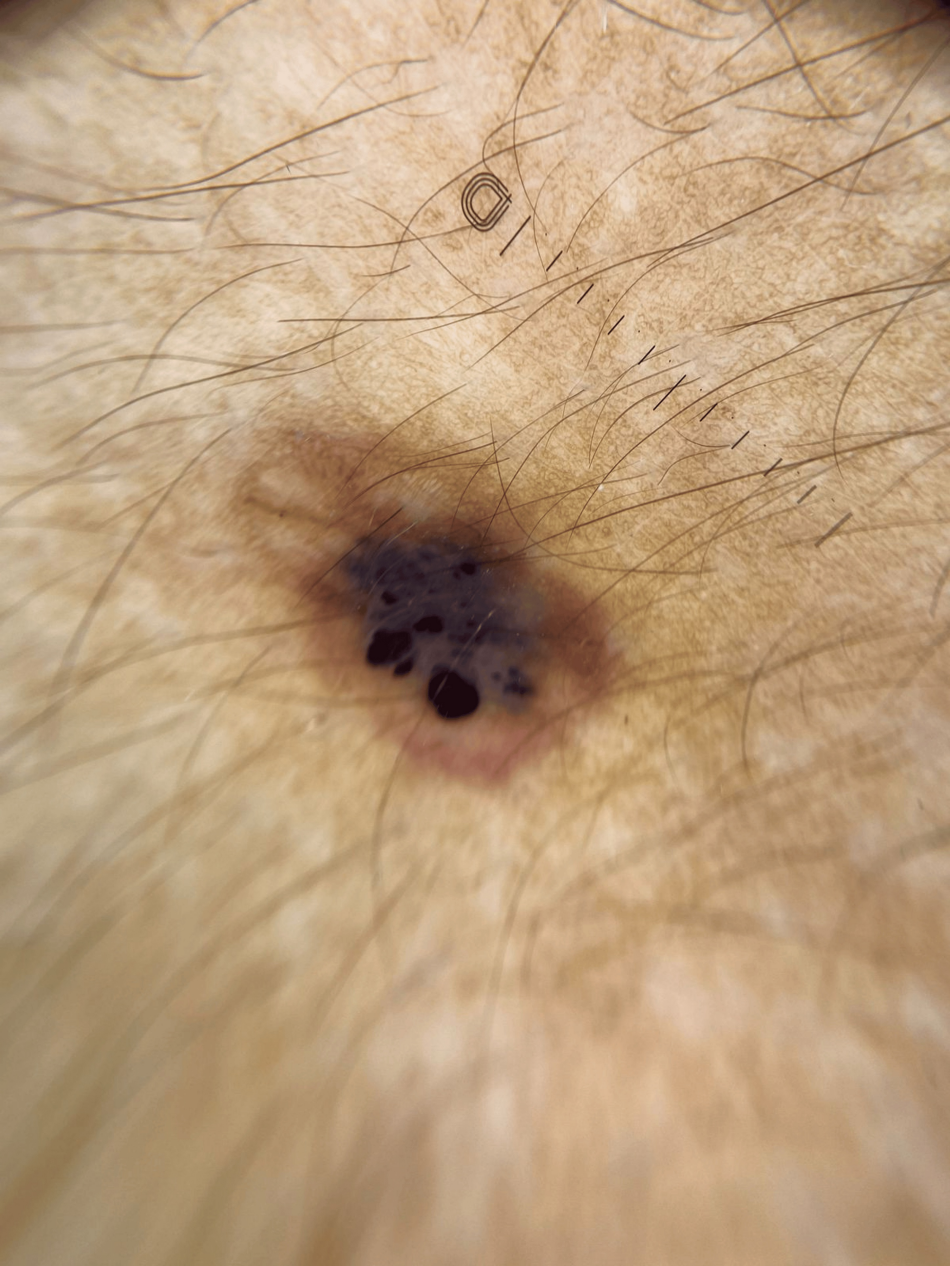
Dermoscopy showing a central blue-black area, which is surrounded and demarcated by a red homogenous area with vascular structures

There was no history of progression in dimension, structure, or color. Further, there was no history of similar lesions on any other body part. No aggravating or relieving factors were reported. There was no sign or symptom suggestive of a history of pain, itching, or tenderness on or around the lesion. Malignant conditions were ruled out based on the lack of complaints consistent with the patient's history. The patient did not have a history of any chronic infections or addictions. There was no family history of skin lesions or any dermatological complaints. The patient was completely normal and could perform all activities expected out of a person of his age. A dermoscopic examination showed a central blue-black area, which is surrounded and demarcated by a red homogenous area with vascular structures. The central lesion is margined by red borders through its circumference (Figure 2).

**Figure 3:**
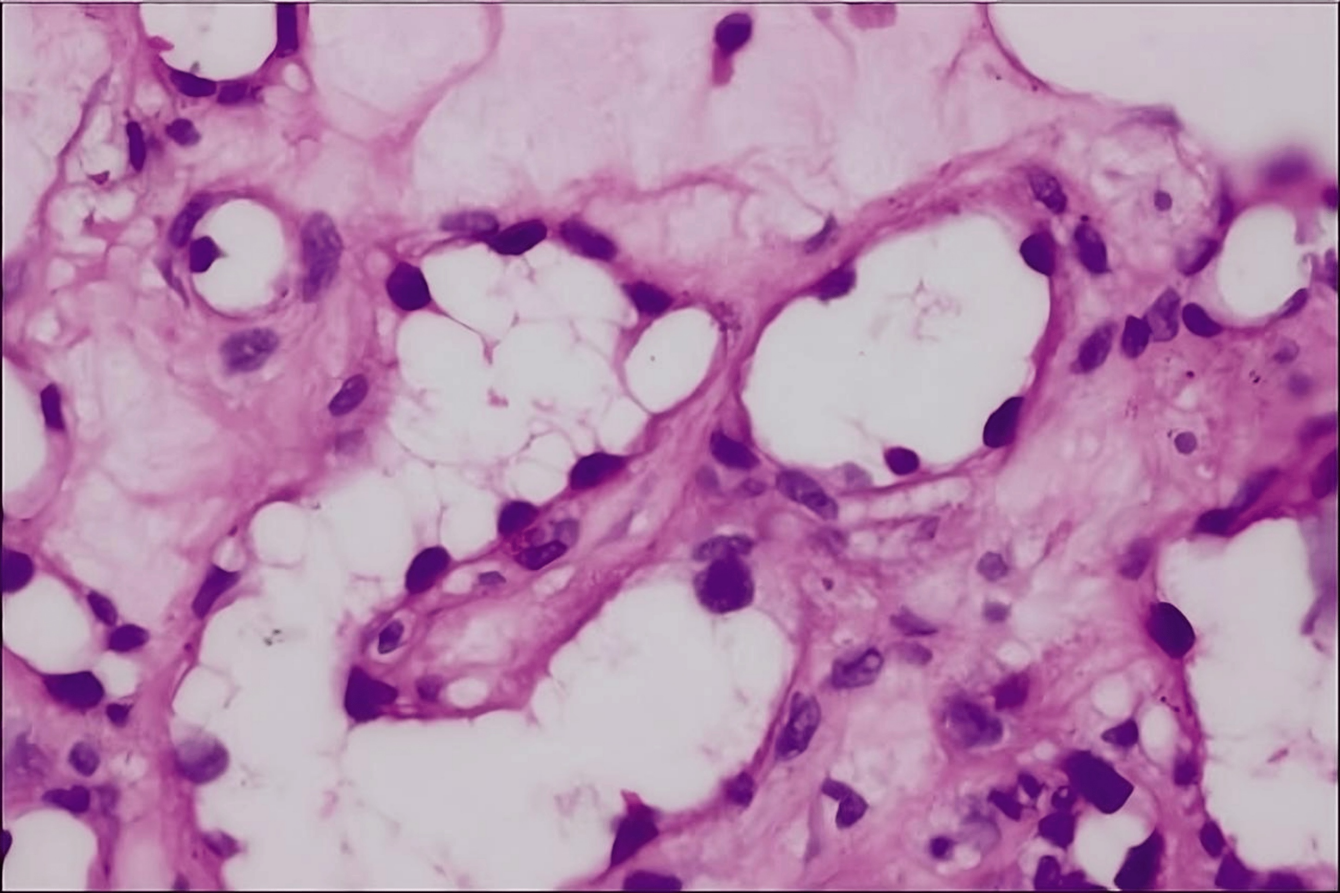
Histopathological examination revealing dilated blood vessels lined by hobnail epithelial cells (H&E ×400) H&E: hematoxylin and eosin

An excisional biopsy was performed on the patient for histopathological examination, which revealed dilated blood vessels lined by hobnail epithelial cells along with hemosiderin depositions in the deeper dermis (Figure 3).

Based on presenting history, contact dermoscopy, and biopsy, the diagnosis of THH was made. The biopsy in itself was curative in addition to being a key factor in the establishment of a definitive diagnosis.

## Discussion

Potential differential diagnoses include insect bite, dermatofibroma, infantile hemangioma, tufted angioma, melanocytic lesions, and Kaposi sarcoma, based on its current stage of evolution. The differential diagnosis includes early Kaposi's sarcoma, epithelioid hemangioma, progressive lymphangioma, and well-differentiated angiosarcoma based on presenting histology. In late lesions, fibrosis, profuse hemosiderin, and collapsed vascular lumina have all been documented [5]. Injury is proposed as one of the primary reasons for the targetoid appearance, even though the exact etiology of THH has not been determined. Trauma can cause the creation of microshunts, in which the capillary pressure causes erythrocytes to fill the lymph spaces within the lesion, hence encouraging the development of aneurysmal microstructures. If specific efferent lymphatic veins are occluded, there will be inflammation, fibrosis, and interstitial hemosiderin deposits [6].

Histologically speaking, it is an abundance of uncircumscribed veins that may penetrate subcutaneous tissue. A superficial proliferation of ectatic dermal vascular lumina with intraluminal papillary projections seems to be the earliest discovery. Endothelial cells have strong intraluminal projections and are either flat or distinctly epithelioid. The angular, lymphatic-like lumina that cluster around sweat gland coils and frequently form tiny hemangiomatous nodules comprise the deeper component. Widespread fibrin thrombi, inflammatory aggregation, and red cell extravasation are seen [7]. Later phases show a considerable buildup of stromal hemosiderin. In endothelial cells, Ulex europaeus 1 lectin is considerably positive, whereas factor VIII-related antigen is just marginally positive. While self-limited, the lesion appears to be irreversible. Although it appears benign clinically, its histologic features are alarming [8]. The clinical-pathological connection serves as the foundation for diagnosis, particularly when it comes to the distinctive shape of the lesion. A straightforward excision can be used to make a correct histological diagnosis and is also therapeutic [9]. Since there is no evidence of local invasion or dissemination, such lesions are simply excised for diagnostic or cosmetological reasons due to the benign nature of the condition [10].

## Conclusions

THH is a relatively uncommon benign disease that histologically resembles a malignant condition. Depending on the developmental stage at which the lesion was biopsied, histology varies. Early phases have a biphasic pattern: in the papillary dermis, tiny hemangiomatous nodules that split the collagen bundles are often formed by vascular spaces that are dilated and slit-like, resembling lymphatic veins that are concentrated near sweat glands. Similar to lymphatic vessels, the vascular compartments in the deep dermis are angulated, slit-like, have solid intraluminal projections, and resemble hobnails. Later stages reveal considerable hemosiderin buildup in the stroma with fibrosis in addition to a collapsed artery lumen. Despite its well-documented histology, it is unclear whether this tumor is caused by endothelial cells in blood arteries or lymphatic vessels. The diagnosis is based on a clinical-pathological connection, notably the lesion's unique appearance. Curative simple excision allows for an accurate histological diagnosis. There have been no confirmed recurrences since the lesion was removed. To ensure an early diagnosis, extensive research of the disease's atypical manifestations and potential treatment choices is required. As a result, the diagnosis is unusual and should be considered. Patients should feel reassured because the illness in itself is generally benign and has a good prognosis for treatment.
